# Treatment, Monitoring, and Management of Patients With Relapsed/Refractory Chronic Lymphocytic Leukemia and Mantle Cell Lymphoma: Evidence From a Multi‐Country Needs Assessment

**DOI:** 10.1002/cnr2.70655

**Published:** 2026-08-13

**Authors:** Patrice Lazure, Toby A. Eyre, Carsten Utoft Niemann, Nirav N. Shah, Martin Dreyling, Florence Cymbalista, Sophie Peloquin, Serge Znamensky, Benjamin Goebel, Suzanne Murray

**Affiliations:** ^1^ AXDEV Group Inc Montreal Canada; ^2^ Oxford University Hospitals Oxford UK; ^3^ Department of Clinical Medicine University of Copenhagen Copenhagen Denmark; ^4^ Department of Hematology Rigshospitalet Copenhagen Denmark; ^5^ Santa Lúcia Hospital Brasília Brazil; ^6^ Medical College of Wisconsin Milwaukee Wisconsin USA; ^7^ Ludwig‐Maximilians‐University Hospital Munich Munich Germany; ^8^ Avicenne Hospital Bobigny France; ^9^ Lilly Oncology Brussels Belgium; ^10^ Eli Lilly and Company Bad Homburg Germany

**Keywords:** clinical management, continuing medical education, hematological cancer, leukemia, lymphoma, needs assessment

## Abstract

**Background:**

The treatment landscape for relapse/refractory chronic lymphocytic leukemia (CLL) and mantle cell lymphoma (MCL) has evolved with recent novel agents. With the availability of novel agents that slow disease progression, managing treatment side effects and monitoring response in patients with these hematologic malignancies may present a challenge for healthcare providers. The imperative to engage patients in the management of their condition demands that specialists are knowledgeable about new treatment options and are skilled and confident in educating patients to manage and monitor for potential side effects.

**Aims:**

This study aimed to establish the current knowledge, skills and confidence levels of oncologists, hematologists and hematologist‐oncologists (ONC/HEM) and identify the challenges they experience when managing and monitoring treatment and engaging patients with CLL or MCL in shared decision‐making. The long‐term aim is to inform evidence‐based continuing medical education to optimize ONC/HEM competencies.

**Methods and Results:**

This cross‐sectional study employed a quantitative survey, completed by 285 ONC/HEM practicing in academic or community settings in Brazil, France, Germany, the United Kingdom, and the United States. Using 5‐point Likert‐type scales, participants reported their knowledge/skills/confidence levels, and their agreement with selected statements. Sub‐optimal levels of knowledge/skills to identify and manage side effects associated with treatments for CLL or MCL were reported. Challenges with patient risk stratification and engaging patients in shared decision‐making were reported, with a greater proportion reported by those in community settings. Of note, 31% of participants reported sub‐optimal skills identifying patients at higher risk for progression and using stratification to plan and manage treatment, and 58% never/rarely/sometimes considered patients' concerns about treatment safety.

**Conclusion:**

Continuing medical education activities to improve knowledge of new treatments and associated pathways for patients with CLL or MCL, and to develop skills/confidence to engage in shared decision‐making are recommended. Setting‐specific issues should be integrated into interventions to ensure relevant educational support for the ONC/HEM.

## 1 Introduction

Chronic lymphocytic leukemia (CLL) and mantle cell lymphoma (MCL) are complex, heterogeneous lymphoid malignancies. CLL is a common form of leukemia, affecting an estimated 4.9 out of 100 000 people in the United States (US), and 1–2/100 000 in Europe per year, and has a clinical course that may deviate significantly depending on results of genetic testing and the identification of certain chromosomal alterations [1, 2]. In MCL, biological (KI‐67) and molecular risk factors (p53) should be similarly determined. Treatment planning for MCL (including blastoid/pleomorphic variants) and to a lesser extent for CLL may therefore rely on molecular testing, including tests detecting mutations which may influence probability of resistance to covalent Bruton tyrosine kinase inhibitors (BTKi) [3].

Patients with CLL typically receive a diagnosis after the age of 70, and those with MCL in their mid‐60s, and both commonly have comorbidities that may impact treatment decisions [4, 5]. Guidelines for the treatment of CLL and MCL [5, 6, 7] recommend stratification of patients based on TP53 mutation [8]/del(17p) status, IGHV mutational stage (CLL only) [9], age, and comorbidities. CLL, regardless of del(17p) or TP53 status, can be treated with targeted agents (acalabrutinib, ibrutinib, zanubrutinib, venetoclax, or a BTKi‐venetoclax combination) [10, 11, 12]. For CLL that is IGHV‐mutated and TP53 wild‐type (no del(17p) or TP53 mutations), chemoimmunotherapy was an alternative option at the time this study was conducted [13]. Comorbidity, frailty and older age may lead to preference for a particular treatment option, taking into account the risk of cardiovascular events with BTK inhibitors, tumor lysis with BCL2 inhibitors and immunosuppression from combinations with CD20 monoclonal antibodies [14, 15].

Guidelines for MCL [1, 7, 16] recommend combination immunochemotherapies ± ibrutinib, with optional autologous stem cell transplantation in younger patients [17]. Molecular markers (e.g., blastoid variant, p53, Ki‐67), age, and fitness are used for risk stratification, which informs the selection of a more or less intensive regimen/therapy [18, 19].

Some patients will have relapse (recurring after remission) or refractory (treatment resistant) CLL or MCL. In these cases, treatment options are further limited, but effective new therapies are being used or are awaiting approval. For CLL these include BTKi (ibrutinib and acalabrutinib), β‐cell lymphoma 2 inhibitors (BCL2i; venetoclax alone or combined with rituximab), idelalisib and rituximab (phosphoinositide 3‐kinase inhibitors) and anti‐CD20 antibodies [20, 21, 22]. Covalent BTKi are options for patients who have been treated with venetoclax, and non‐covalent BTKi (pirtobrutinib) is a potential option for patients after covalent BTKi exposure (e.g., ibrutinib, acalabrutinib, or zanubrutinib). For patients with MCL with early relapse within 24 months after first‐line chemotherapy, a BTKi is typically recommended (EU: ibrutinib, acalabrutinib; US: acalabrutinib, zanubrutinib); alternatively, pirtobrutinib or CAR‐T cells are third‐line options after BTKi failure [23].

Current guidelines describe care pathways that involve hematologists and hematologist‐oncologists as key care providers for those with CLL or MCL [6, 24]. However, not all patients have access to these specialists for a variety of reasons—location and proximity to academic centers, insurance coverage [25, 26, 27]. Adapting to these limitations has underscored the importance of communication and patient engagement.

The crucial role of risk stratification when determining a treatment pathway is understood; however applying these practices is a known challenge, as there are many necessary considerations when selecting appropriate treatment: genetic test results, patient age, comorbidities, treatment history, and efficacy [28]. The significant and complex risk of toxicities and side effects of many treatment options demands precise decision‐making and monitoring whether administered for a fixed or a continuous duration [2, 29].

Oncologists, hematologists and hematologist‐oncologists (ONC/HEM) must balance issues of patient quality of life and adherence, with foresight regarding longer‐term efficacy and risk avoidance. Support is needed to address known deficits in patient education and engagement [30], as optimizing a patient's quality of life demands that they take an active role in their monitoring and treatment decision making. This entails recognizing and reporting side effects and suspected toxicities based on adequate knowledge of their condition, and understanding likely outcomes of care [31].

Recent studies suggest that there is a need to improve provider education and evidence‐based practice in relation to patients with CLL or MCL [32]. Patient outcomes are positively associated with (among other factors) the availability of practice guidelines and access to cancer care centers in their region that implement best practices and ensure adequate quality of care [33]. While clinical data emerge to support novel therapies and combination strategies, guidelines struggle to provide updated knowledge of how to translate this into practice. Additionally, the degree to which the challenges are observed in different countries is less understood [34].

This exploratory cross‐sectional study aims to assess knowledge, skills, and confidence of ONC/HEM with the intention of informing initiatives (continuing education or other types of interventions) to address any identified gaps in the care of patients with relapse/refractory CLL or MCL. Specifically, this study aims to understand gaps in knowledge, skills, and confidence when incorporating new treatments (monitoring and management of side effects), when considering complex patient profiles (risk stratification) and when engaging patients (including using shared decision‐making and considering patient preferences). An additional objective is to quantify country‐ and setting‐specific details to add context to known challenges.

## 2 Methods

This cross‐sectional study employed a survey to collect quantitative data, measuring the prevalence of known or suspected challenges. Using a purposive sampling [35] approach, targeted participants were specialized physicians (i.e., oncologists, hematologist‐oncologists, and hematologists) whose scope of practice includes relapse/refractory CLL and MCL, and whose proficiency in the field has direct impact on patients with these conditions [36] according to guidelines and recommended care pathways [17, 37]. The survey items were developed by educational subject matter experts (co‐authors PL, SP, SM) based on a review of current literature on the challenges associated with monitoring and managing patients with relapse/refractory CLL or MCL. Clinical experts (co‐authors T.A.E., C.U.N., N.N.S., M.D., F.C.) reviewed the survey items for face validity [38, 39]. Reporting of this study follows the CROSS (Consensus‐Based Checklist for Reporting of Survey Studies) reporting guidelines [40](see Supporting Information A).

The survey queried participants about their proficiencies in treating, managing, monitoring, communicating, and collaborating with patients with CLL or MCL, according to what is expected of them in their professional role. Using a five‐point, Likert‐type scale, survey items measured knowledge and skill (1‐none to 5‐expert), frequency (1‐never to 5‐always) and confidence levels (1‐not at all to 5‐very confident). In addition, agreement with given statements was measured using a six‐point Likert scale (1‐strongly disagree to 6‐strongly agree). Some questions were shown only to specific sub‐groups to maximize relevance, and although questions were mandatory, respondents had the option to select “non‐relevant” when they judged a specific statement as not applying to their current practice. More information about the survey is provided in Supporting Information B.

Participants were first surveyed in Germany, France and the US, followed by a second phase of data collection in Brazil and the UK. Findings from the first phase have been published [41]; this paper adds data from two additional countries and includes analyses of some previously unpublished survey items. Also, the increased sample for participants from community settings allowed for additional analyses. Prior to launch, the survey was beta‐tested by the researchers for function and quality assurance. The survey was initially launched to a small sample of potential participants and survey comments were reviewed to ensure good functioning. As no issue was detected, the research team proceeded with the full launch. Some survey items varied according to participant country, to ensure relevance (e.g., questions about specific national guidelines). It comprised a total of 20 questions, 12 of which had multiple items. The survey included self‐reporting questions on knowledge and skills (five‐point, Likert‐type scale, from 1‐none to 5‐expert), and confidence (five‐point scale, from 1‐not at all to 5‐very confident), as well as agreement items (six‐point, Likert‐type scale, from 1‐strongly disagree to 6‐strongly agree). Save for minor adjustments, the survey was the same in both phases to ensure fair comparison. The survey was developed in English, then translated to French, German and Portuguese, and was hosted on a secured online platform. All participants were recruited as individual healthcare providers (i.e., they did not respond on behalf of their institution) and provided their informed consent prior to participation. Those who completed the survey were financially compensated according to their region and profession, in accordance with best practices in research ethics. Study materials and protocol (including participant compensation) were granted ethics approval by an independent review board (Veritas IRB; Tracking Number: 2022‐2930‐9381‐2), who reviewed the study under ethical guidelines and regulations for Canada (where the research team is located) and for each of the 5 participating countries (Brazil, France, Germany, the United Kingdom, and the United States). Veritas IRB (www.veritasirb.com) is an independent organization accredited by Human Research Accreditation Canada (www.hracanada.org), operating in compliance with international regulations and guidelines, including the declaration of Helsinki [42] and WHO's Operational Guidelines for Ethics Committees that Review Biomedical Research [43]. All study data were securely stored in a password protected database with restricted access.

### 2.1 Participants and Data Collection

Potential participants were recruited from an online panel of physicians who registered, as individuals, to receive invitations for research studies, operating in accordance with the ICC/ESOMAR International Code on Market, Opinion and Social Research and Data Analytics [44]. Potential participants received an email with a link to the online survey, which began with a few eligibility questions. The panel used a unique identifier that prevents the same participant from completing the survey multiple times. The criteria for inclusion in this study were as follows: participants must be oncologists, hematologist‐oncologists, or hematologists, and be actively practicing (≥ 50% of their time in patient care) in France, Germany, Brazil, the UK, or US. Participants must have had 2 years or more post‐residency experience, have treated a minimum of two (MCL) or five (CLL) patients in the last 2 years and have prescribed BTKi to those patients at least once. The final criterion was included to ensure recruitment of participants who were impacted, at least minimally, by the change in the treatment landscape for patients with CLL or MCL that had occurred with the approval of oral targeted therapies such as BTKi [45, 46]. Selection of recipients for the email invitation was based on profession, specialty and country of practice, but other inclusion criteria and interest in CLL or MCL could not be verified prior to the invitation, resulting in large number of invitations and low response rates (defined here as completed surveys/invitations sent). Eligible participants were then presented with the consent form, and upon providing consent, were invited to complete the survey immediately.

Eligible participants were organized into seven main sub‐groups based on country and whether they were practicing in an academic setting (if in France), a community‐based setting (if in Brazil or the UK) or either (if in the US or Germany). These sub‐groups were selected in accordance with each country's health system structure, including only settings that were most likely engaged and responsible for care for patients with CLL or MCL. In France, these conditions are mostly managed in academic settings, while in Brazil and the UK, cancer care and hematology are mostly done in community‐based settings [24, 47]. To the authors' knowledge, care for CLL and MCL in Germany and the US is done in both settings, so participants from both settings were included. Academic settings were defined as hospitals or research centers that are associated or affiliated with a university, and community settings as hospitals, clinics, government medical centers or private practices that are unaffiliated with a university. Stratification based on setting was used to investigate, as some studies suggest, potential differences in each setting's approach to managing side effects, risk stratification, management and monitoring, and communication [48]. Additionally, much care is provided in community settings, yet studies often focus on academic settings [49].

Target sample sizes were determined a priori, based on the educational co‐authors' experience with similar studies [50, 51, 52]. Sample sizes were based on the exploratory nature of the study, and that its aim was to provide a descriptive portrait of current challenges that could serve as a starting point to inform interventions to improve knowledge, skills, and confidence of ONC/HEM involved in the care of patients with relapse/refractory CLL or MCL and not to test a specific hypothesis. Data were collected in the first phase from March 16 to April 29, 2022, and in the second phase from June 22 to July 31, 2022. Purposive sampling was applied during data collection to prevent some potential biases, with regular monitoring to ensure a variety of demographics and clinical characteristics among the sample, in terms of setting, years of experience, location and caseloads [35].

### 2.2 Analysis

Survey responses were grouped to illustrate educational needs (e.g., 1‐no, 2‐basic and 3‐intermediate knowledge/skills; 1‐not at all, 2‐slightly, 3‐somewhat confident are described as “sub‐optimal,” while 4–advanced, 5‐expert knowledge/skills and 4‐confident, 5‐very confident were grouped as “optimal”). Agreement items were grouped to describe sentiment and attitude (4‐slightly to 6‐strongly agree described as “agree,” 1‐strongly disagree to 3‐slightly disagree as “disagree”). For each agreement item, the desirable response (agreement or disagreement) was determined through consultation among members of the project's clinical expert committee (co‐authors T.A.E., C.U.N., N.N.S., M.D., F.C.). Responses of “non‐relevant” were excluded from analysis.

Data were analyzed unweighted, using descriptive statistics (cross‐tabulations and chi‐squares) with IBM SPSS for Windows 27.0 (IBM Corp., Armonk, N.Y., USA), with the aim of synthesizing key findings and drawing conclusions regarding knowledge, skills, and confidence of CLL and MCL treatment options. Findings were subject to contextualization and interpretation by both clinical (co‐authors T.A.E., C.U.N., N.N.S., M.D., F.C.) and educational subject matter experts (co‐authors P.L., S.M., S.P.).

## 3 Results

Table 1 shows demographic data on the oncologists, hematologists, and hematologist‐oncologists (*n* = 285) from academic (*n* = 129, 45%) and community (*n* = 156, 55%) settings who participated in this study, including their country, practice setting, years of experience, and median caseload of CLL and MCL patients. To recruit these participants, invitations were sent to a broad spectrum of oncologists, hematologist‐oncologists, and hematologists (*n* = 8500 for Phase 1; *n* = 7311 for Phase 2), for a completion rate of 1.8% for both phases combined.

**TABLE 1:** Demographic/clinical characteristics of study participants.

Country	US	FR	GER	UK	BR	Total
Practice setting						
Academic	50	57	22	—	—	45% (129)
Community	65	—	30	30	31	55% (156)
Total	115	57	52	30	31	285

Country	US		FR	GER		UK	BR	Total
	ACAD	COM	ACAD	ACAD	COM	COM	COM	
Distribution of respondents per years of experience^a^								
2–5 years	8% (4)	6% (4)	5% (3)	5% (1)	10% (3)	10% (3)	65% (20)	13% (38)
6–10 years	18% (9)	26% (17)	16% (9)	18% (4)	27% (8)	20% (6)	13% (4)	20% (57)
11–20 years	32% (16)	42% (27)	51% (29)	55% (12)	47% (14)	63% (19)	16% (5)	43% (122)
21 years or more	42% (21)	26% (17)	28% (16)	23% (5)	17% (5)	7% (2)	6% (2)	24% (68)
How many patients with mantle cell lymphoma (MCL) have you treated in the past 2 years?								
Median	47	40	32	48	40	27	10	30
25th Percentile	14	14	16	16	10	15	5	12
75th Percentile	318	85	80	69	76	46	20	78
How many patients with chronic lymphocytic leukemia (CLL) have you treated in the past 2 years?								
Median	95	85	95	112	80	70	30	80
25th Percentile	40	37	49	68	40	50	20	40
75th Percentile	423	200	150	200	157	128	50	173

Abbreviations: ACAD, Academic setting; BR, Brazil; COM, Community setting; FR, France; GER, Germany; UK, United Kingdom; US, United States.

^a^ The values don't add to 100% due to rounding.

Results suggest that certain challenges and barriers impact treatment, monitoring, and management for patients with relapse/refractory CLL or MCL. The identified challenges and educational gaps facing the seven sub‐groups are categorized into four themes: 1‐treatment side effects, safety, and efficacy; 2‐monitoring and management of side effects; 3‐risk stratification and treatment adjustment; 4‐patient engagement and shared decision‐making.

### 3.1 Treatment Side Effects, Safety and Efficacy

**FIGURE 1:**
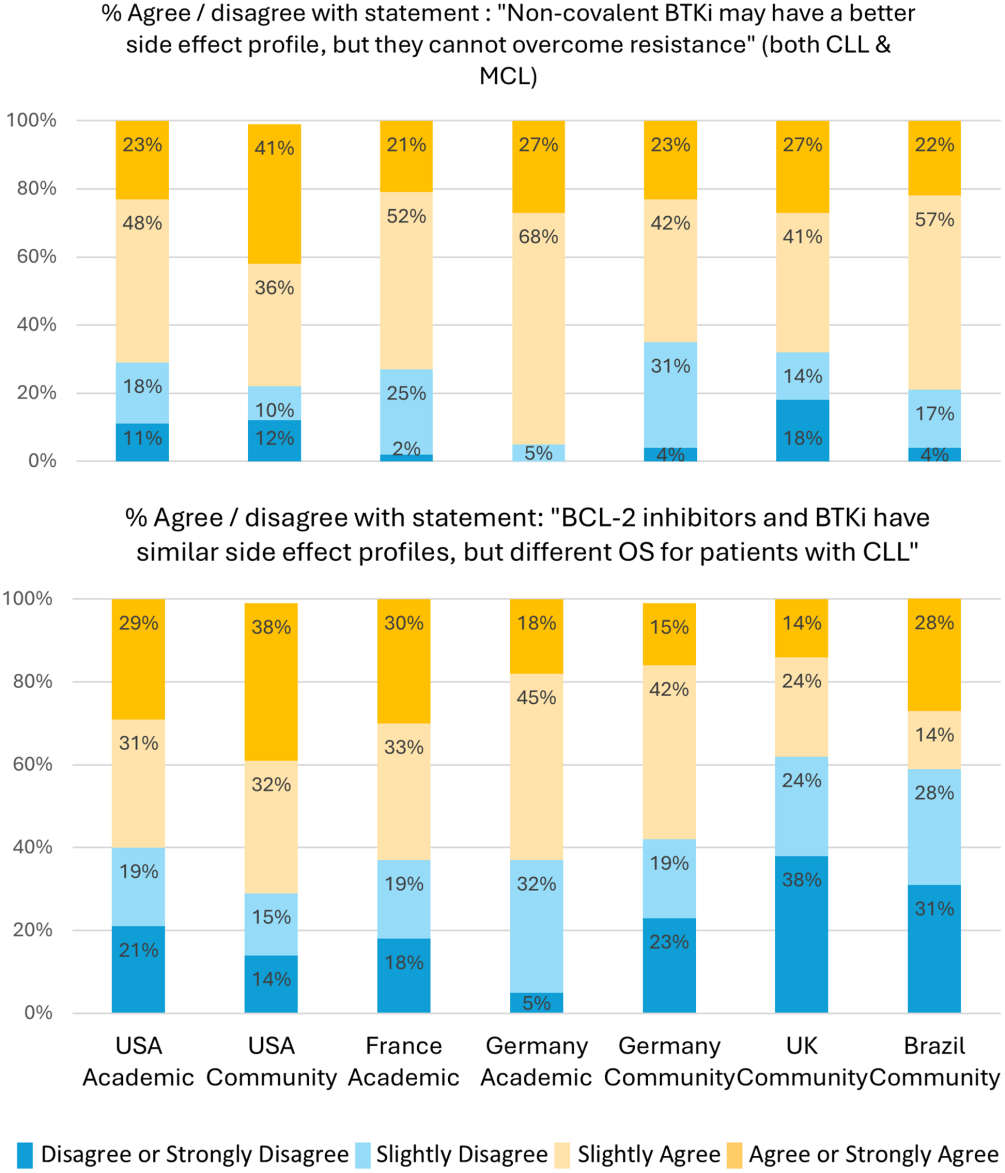
Level of agreement with statements by country and setting: % Agree/disagree with statement: “Non‐covalent BTKi may have a better side effect profile, but they cannot overcome resistance” (both CLL & MCL), % Agree/disagree with statement: “BCL‐2 inhibitors and BTKi have similar side effect profiles, but different OS for patients with CLL.”

**FIGURE 2:**
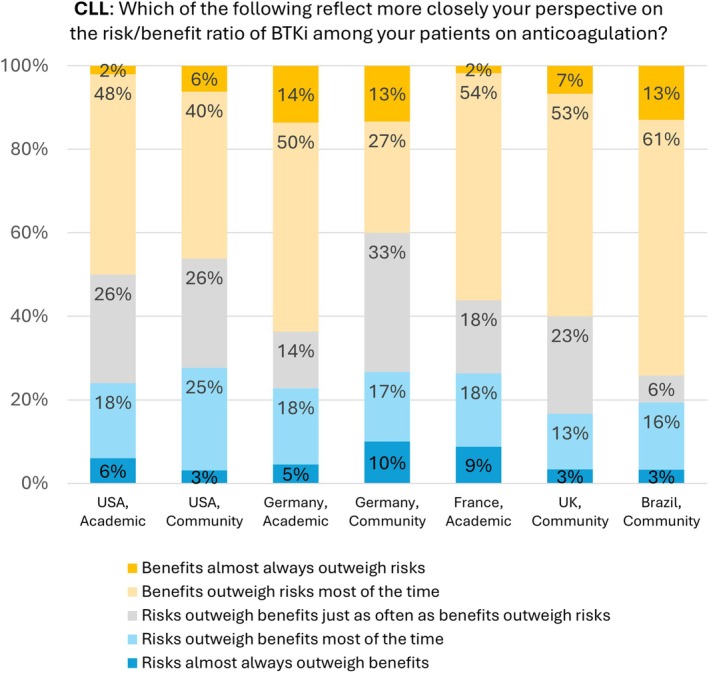
Perspective of risk–benefit balance of BTKi for patients with CLL by country and setting CLL: Which of the following reflects more closely your perspective on the risk/benefit ratio of BTKi among your patients on anticoagulation?.

In CLL, knowledge gaps were identified regarding side effect profiles of recently approved BTKi (34%), and non‐covalent BTKi (49%) with greater gaps in Brazil, 68%, and UK, 70% (see Table 2). An elevated proportion of participants in the US community‐based setting (37% vs. 24% total) reported no, basic, or intermediate (i.e., “sub‐optimal”–see methods) knowledge of the side effect profiles of CD20 monoclonal antibodies (e.g., rituximab, obinutuzumab). A divided understanding of the risks involved in treatment was observed, with half (49%) of all respondents disagreeing with the statement “BCL‐2 inhibitors and BTKi have similar side effect profiles, but different OS for patients with CLL,” with considerably higher disagreement levels in UK and Brazil than in other countries (see Figure 1, bottom panel). Additionally, a lack of consensus on risk: benefit ratio of BTKi for those with CLL on anticoagulants was reported, with 25% indicating that the risks of BTKi for patients with CLL on anticoagulants outweigh benefits “most of the time” or “almost always,” while an additional 22% believe risks outweigh benefits just as often as benefits outweigh risks (Figure 2).

**TABLE 2:** % (*n*) reporting sub‐optimal knowledge^a^.

Knowledge item side effect profiles of	US		FR	GER		UK	BR	Total
	ACAD	COM	ACAD	ACAD	COM	COM	COM	
Non‐covalent BTK inhibitor (CLL)	39% (19)	44% (28)	47% (27)	48% (10)	40% (12)	70% (21)	68% (21)	49% (138)
CD20 mono‐clonal antibodies (CLL)	21% (10)	37% (23)	21% (12)	24% (5)	30% (9)	20% (6)	6% (2)	24% (67)
Recently approved (after 2018) BTKi (CLL)	34% (16)	38% (24)	37% (21)	24% (5)	33% (10)	27% (8)	32% (10)	34% (94)

Abbreviations: ACAD, Academic setting; BR, Brazil; COM, Community setting; FR, France; GER, Germany; UK, United Kingdom; US, United States.

^a^ Sub‐optimal knowledge = % (*n*) who reported 1‐no, 2‐ basic, or 3‐intermediate knowledge when asked to rate their knowledge level for a given item.

### 3.2 Monitoring and Management of Treatment Side Effects

Over half (54%) of participants reported sub‐optimal skill levels (i.e., no, basic, or intermediate) when monitoring and managing side effects from CAR‐T cell therapy in MCL with a greater gap in Germany (72%) and Brazil (77%) community settings, and nearly one‐third (32%) when it comes to BCL‐2 and BTKi for CLL (Table 3). A greater skill gap was found in the German community‐based setting for BCL‐2 (43%), and in the French academic setting for BTKi (40%). In the US academic setting, 32% of participants reported themselves as not, slightly, or moderately confident when selecting treatment for both MCL (compared to 24% total) and CLL (compared to 17% total) in consideration of potential side effects and toxicities.

**TABLE 3:** % (*n*) reporting sub‐optimal skills^a^.

Skills item	US		FR	GER		UK	BR	Total
	ACAD	COM	ACAD	ACAD	COM	COM	COM	
Monitor and manage side effects from BCL‐2 inhibitors (CLL)	29% (14)	37% (23)	37% (21)	27% (6)	43% (13)	27% (8)	16% (5)	32% (90)
Monitor and manage side effects from BTKi (CLL)	29% (14)	38% (25)	40% (23)	23% (5)	33% (10)	23% (7)	19% (6)	32% (90)
Monitor and manage side effects from BTKi (MCL)	25% (12)	37% (24)	39% (22)	9% (2)	37% (11)	23% (7)	23% (7)	30% (85)
Monitoring and managing side effects from CAR‐T cell therapy (MCL)	40% (19)	44% (28)	53% (30)	52% (11)	72% (21)	53% (16)	77% (24)	54% (149)
Identifying patients at higher risk for progression and using stratification to plan and manage treatment (CLL)	28% (14)	35% (23)	33% (19)	27% (6)	43% (13)	30% (9)	13% (4)	31% (88)
Weighing guideline recommendation and patient preferences to select new treatment in case of tolerance or toxicity (CLL)	32% (16)	32% (20)	36% (20)	32% (7)	50% (15)	33% (10)	20% (6)	33% (94)
Weighing recommendations from guidelines and patient preferences to select new treatment in case of tolerance or toxicity (MCL)	—	—	53% (30)	23% (5)	43% (13)	50% (15)	26% (8)	42% (71)
Incorporating recommendations from guidelines to adjust treatment (CLL)	36% (18)	37% (24)	32% (18)	27% (6)	40% (12)	27% (8)	10% (3)	31% (89)
Incorporating recommendations from guidelines to adjust treatment (MCL)	25% (12)	33% (21)	—	—	—	43% (13)	35% (11)	33% (57)
Adjusting treatment approach based on patients' tolerance (CLL)	33% (16)	31% (20)	23% (13)	32% (7)	40% (12)	27% (8)	10% (3)	28% (79)

Abbreviations: ACAD, Academic setting; BR, Brazil; COM, Community setting; FR, France; GER, Germany; UK, United Kingdom; US, United States.

^a^ Sub‐optimal skills = % (*n*) who reported 1‐no, 2‐basic, or 3‐intermediate skills when asked to rate their skill level for a given item.

### 3.3 Risk Stratification and Treatment Adjustment

Sub‐optimal skills were reported when identifying patients at higher risk for tumor progression and using stratification to plan and manage treatment by 31% of participants overall, and by a relatively higher proportion (43%) of those in community‐based settings in Germany. A third (33%) of participants reported sub‐optimal skill levels when weighing patient preferences with guideline recommendations to select a new course of treatment (50% German community‐based settings). Sub‐optimal skill levels were also reported when selecting treatment in the presence of intolerance or toxicity (42% overall, 53% in France, 50% UK). When adjusting treatment, 40% of those in German community‐based settings report sub‐optimal skills when incorporating recommendations from guidelines (31% total) and when making those adjustments based on patient tolerance (28% overall, see Table 3).

In all countries, 28% reported sub‐optimal confidence in recognizing when patients develop resistance to BTKi. In Brazil 42% reported sub‐optimal confidence. Data suggests that BTKi are perceived to have limitations: 28% agree/strongly agree, and 47% slightly agree with the statement: non‐covalent BTKi may have a better side effect profile, but they cannot overcome resistance (See Table 4 and Figure 1).

**TABLE 4:** % (*n*) reporting sub‐optimal confidence^a^.

Confidence item	US		FR	GER		UK	BR	Total
	ACAD	COM	ACAD	ACAD	COM	COM	COM	
Selecting treatment in consideration of potential side effects and toxicities (CLL)	32% (16)	17% (11)	12% (7)	18% (4)	13% (4)	10% (3)	6% (2)	17% (47)
Selecting treatment in consideration of potential side effects and toxicities (MCL)	32% (16)	27% (17)	23% (13)	18% (4)	27% (8)	17% (5)	13% (4)	24% (67)
Engaging patients in discussion of risk and benefits of therapy including side effects, overall survival (OS), and progression free survival (both CLL & MCL)	14% (7)	26% (17)	28% (16)	14% (3)	20% (6)	17% (5)	16% (5)	21% (59)
Educating patients to help them distinguish what is a lack of response to treatment vs. a manageable side effect (both CLL & MCL)	29% (14)	25% (16)	19% (11)	18% (4)	27% (8)	30% (9)	16% (5)	24% (67)
Recognizing BTK inhibitor resistance (both CLL & MCL)	18% (9)	26% (16)	28% (16)	27% (6)	30% (9)	37% (11)	42% (13)	28% (80)

Abbreviations: ACAD, Academic setting; BR, Brazil; COM, Community setting; FR, France; GER, Germany; UK, United Kingdom; US, United States.

^a^ Sub‐optimal confidence = % (*n*) who reported 1‐not, 2‐slightly, or 3‐somewhat confident when asked to rate their confidence level for a given item.

### 3.4 Patient Engagement and Shared Decision‐Making

**FIGURE 3:**
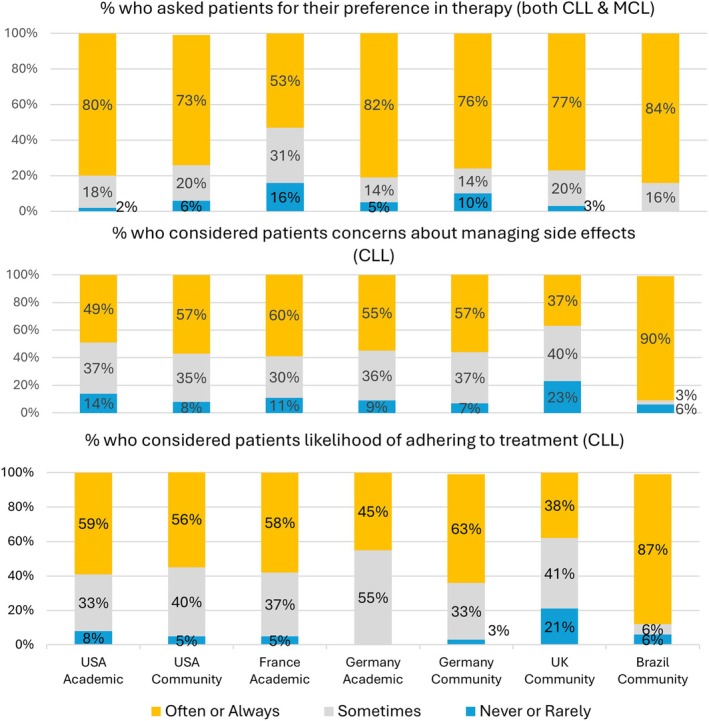
Frequency of tasks performed by country and setting: % who asked patients for their preference in therapy (both CLL & MCL), % who considered patients concerns about managing side effects (CLL), % who considered patients likelihood of adhering to treatment (CLL).

Participants reported infrequency in addressing many of the patient concerns surveyed. Of note, respondents stated that they never, rarely, or only sometimes asked patients about their preferences in therapy (25% total, and 47% in France); or considered either patients' concerns about managing side effects or their likelihood of adhering to the treatment (each 42% total) (See Figure 3).

Certain aspects of patient education and support in shared decision‐making were found to be challenging. The engagement level and confidence to discuss issues of treatment safety and prognosis were low. Of all respondents, 58% never, rarely, or sometimes considered patients' concerns about treatment safety—lack of evidence, and 21% lacked confidence engaging patients in discussion of risks and benefits of therapy including side effects, overall survival (OS), and progression free survival (PFS) (highest in France 28%, and German academic 26%) (See Table 4 and Figure 3).

Confidence was sub‐optimal when educating patients to help them distinguish what is a lack of response to treatment vs. a manageable side effect among those in academic settings in the US (29%), and in community‐based settings in Germany (30%), (28% of total) (See Table 4).

## 4 Discussion

This study explored the challenges and barriers to providing care for patients with CLL or MCL, with special attention to variables such as participants' country and practice setting. Investigations into these issues are increasing. A recent US study explored education gaps of CLL and MCL professionals [53], while another reported divergent approaches to (and outcomes of) treatment selection and care practices between academic and community settings [54, 55]. This report adds to existing literature by providing an international perspective and a gap‐oriented approach to supplement these recent studies. Key gaps were identified in four areas: (1) treatment side effects, safety, and efficacy; (2) monitoring and management of side effects; (3) risk stratification and treatment adjustment; and (4) patient engagement and shared decision‐making. These findings complement the results obtained from the previously published first phase [41] by providing additional analyses based on respondents' practice settings and uncovering a theme that was not prioritized with the smaller sample, patient engagement and shared decision‐making.

In France and in community‐based settings in Germany and the US in particular, the data suggest a concerning level of participants who perceive BCL‐2i and BTKi to have similar side effect profiles but different OS for CLL patients [56]. This, combined with a lack of consensus on the perceived risk of treating patients who use anticoagulants with BTKi, despite evidence supporting their safe use [57], indicates a need for educational intervention on these impactful facets of treatment decision making.

Low skill levels were reported when monitoring and managing side effects of BCL‐2i and BTKi, and CAR‐T cell therapy, by all except those in UK and Brazil. One explanation may be the approval status and/or access to these therapies in practice, and the degree of continuing education available to providers in anticipation of their use. ONC/HEM's confidence to select treatment is impacted by proper access to novel therapies and can be supported not only by relevant and up‐to‐date guidelines, but by efficient implementation strategies of these guidelines and best practices [58].

Multiple factors may explain differences observed between countries and settings. Globally, higher‐income countries are associated with fewer barriers related to wait time and financial concerns, and less barriers to latest treatments overall [59]. Regulations and population disease burden are factors to consider as national variables, and system‐specific issues such as divergent institutional resources and practices are relevant to community and academic settings. For example, CAR‐T cell therapy has been approved for certain leukemias as early as 2017 in the US, and only in the past year in Brazil. In response to European approval in 2018, the real implications of delivering these therapies exposed new potential challenges—securing qualified centers, manufacturers and compliance with safety regulations [60]. Approval of a novel therapy may occur long before it is integrated into national or international guidelines, institutional protocol, and clinical prescribing practices. Depending on a country's health system, there may be a discrepancy between when public reimbursement and/or recommendations are available and initial regulatory approval [25]. It follows that academic centers may benefit from better access to clinical trials for improved familiarity and confidence using new therapies, while community settings await access.

Addressing the challenges suggested by our study could potentially contribute to optimizing patient management and monitoring that may be complicated by the providers' resources and/or access to specialists. As Jain [3] outlines, predicting BTKi resistance and determining how best to manage patients who progress on BTK/BCL‐2 inhibitors, as well as factoring the patient's genomic profile into treatment are enduring issues in this field [3]. Also, factoring in secondary immune deficiency and the risk of toxicity versus efficacy on different treatment options is an ongoing challenge [61]. Our study uncovered a perception that the side effects of BTKi and other therapies are severe or difficult to manage—improving knowledge in this area will facilitate the confidence and skills needed to manage patients within this novel treatment landscape, as well as effectively communicate and build a collaborative approach with patients as partners in their care. This would also include taking comorbidities and concomitant medications with anticoagulants and other risk factors into account when assessing safety and efficacy for individual patients [62].

This study highlights potential challenges hindering providers' aim to meet the needs of patients who are being treated with BTKi. Patients have indicated a desire for education and support in self‐management practices [63]. Though patients do report positive experiences with ibrutinib, acalabrutinib, and venetoclax, they also experience significant side effects [64]. Side effect management and communication and collaboration are known areas where ONC/HEM and other providers struggle [65, 66]. Communication with patients to encourage disclosure of those side effects and how they impact their experience and quality of life is important, as are the sometimes neglected discussions of patient preferences [67]. These topics should be approached in an effort to ensure adherence to treatment, which is key for this patient population who sometimes require continuous therapy.

This study emphasizes the enduring challenges in this area. We found that the HCPs most involved in these patients' care still struggle with low confidence in their ability to provide patient education and support. In short, there is a need to improve awareness of the benefits of involving patients in care, gain skills to provide that support and implement tools for engagement. One approach is to develop and improve ONCs'/HEMs' proficiencies as educators and provide practical training for them to engage in shared decision‐making—a technique that has been successful in increasing patient experience and outcomes [68].

### 4.1 Implications of the Study

The results of this study suggest a need for educational interventions and activities to build skills and confidence of HCPs in engaging patients in shared decision‐making, and to ensure in‐depth working knowledge of the side effect profiles of existing and new treatments. Additionally, it points to the need for physician training to better incorporate patient decision making in treatment recommendations given the variety of options available for these diseases. In particular, CME initiatives focusing on treatment approaches could help learners develop skills needed to engage patients with complex profiles (those with relapse/refractory CLL or MCL, those with comorbidities or secondary immune deficiency) with the intention to provide a standard of care across settings and countries. Lastly, this survey highlighted a potential lack of patient involvement in decision making. Given the multitude of options available for both CLL and MCL, each treatment which leads to an individual patient journey, additional CME targeting ONC/HEM providers is needed to ensure treatment decisions are in line with patient needs and preferences. Such CME initiatives should be developed considering the local context, including access and approval status of the treatment options, as well as CME requirements and regulations.

### 4.2 Limitations of the Study

The perspectives of those who are adjacent to the target population (e.g., patients, oncology nurses) of this study were not included, but would provide a more comprehensive understanding of the subject in future studies. Similarly, inclusion of qualitative data could have strengthened findings and improved identification of causal factors and potential solutions. The study had a relatively small sample size (1.8% overall response rate), due to limited capacity to specifically target providers with an established interest in CLL or MCL, and to the difficulty of reaching out to target populations in academic settings in targeted countries. This limits the ability to provide specific recommendations based on the variables investigated. Recruitment biases cannot fully be excluded, as physicians who registered to receive research study invitations from an online panel might differ from the general population of physicians. As the study was not designed to provide statistical power to compare results across countries, the results and comparisons should be interpreted as purely descriptive in nature. In addition, the survey relies on self‐reported knowledge, skill and confidence, and may not fully reflect objectively assessed competence or actual practice. Certain demographic features (e.g., practicing in private versus public healthcare systems) were not included in study design. Finally, some questions were asked considering both CLL and MCL, which may have yielded different responses if asked separately for each condition.

## 5 Conclusion

This study sought to approach education gaps in MCL and CLL in greater depth and breadth especially in light of emerging novel therapeutics, new guideline recommendations on risk stratification, and evolutions in patient care approaches that favor individualized and shared decision‐making practices. Gaps in knowledge and skills were observed among providers, in areas of importance to patient care: selecting, managing and monitoring treatment, and confidence levels were less than optimal when communicating with patients and supporting involvement in their own care. Elevated challenges among those in community settings in many cases, and variation between countries were reported. These factors should be used as guidance for future continuing medical education or continuing professional development initiatives.

## Author Contributions

**Patrice Lazure:** conceptualization, funding acquisition, methodology, supervision, investigation, formal analysis, data curation, writing – original draft. **Toby A. Eyre:** validation, methodology, writing – review and editing. **Carsten Utoft Niemann:** validation, methodology, writing – review and editing. **Nirav N. Shah:** validation, methodology, writing – review and editing. **Martin Dreyling:** validation, methodology, writing – review and editing. **Florence Cymbalista:** validation, methodology, writing – review and editing. **Sophie Peloquin:** conceptualization, funding acquisition, methodology, formal analysis, supervision, investigation, writing – original draft. **Serge Znamensky:** conceptualization, funding acquisition, writing – review and editing. **Benjamin Goebel:** conceptualization, funding acquisition, writing – review and editing. **Suzanne Murray:** conceptualization, funding acquisition, methodology, writing – review and editing.

## Funding

This study was financially supported by education research funds from Eli Lilly & Company to AXDEV Global Inc. Representatives of the funder’s medical education department (co‐author SZ and BG) contributed to the initial conceptualization of the study, the interpretation of aggregated data, and critically reviewed the draft of this article. The funder had no role in the data collection and analysis of the data.

## Ethics Statement

This study and all study materials were granted ethics approval by Veritas IRB (Montreal, QC, Canada; Project tracking Number: 2022‐2930‐9381‐2).

## Conflicts of Interest

P.L. and S.P. are employees of AXDEV Group Inc. S.M. is CEO & founder of AXDEV Group Inc., AXDEV Global Inc., and AXDEV Europe GmbH. T.A.E. has received honorarium from Roche, Gilead, Janssen, AbbVie, AstraZeneca and BMS; Advisory Board honorarium from Roche, KITE, Loxo Oncology, Beigene, Incyte, Autolus, Galapagos, and BMS; speaker honorarium from Incyte, Medscape, PeerView, Clinical Care Options, The Limbic; research support/funding from Gilead, AstraZeneca, and Beigene; travel to scientific conferences from Gilead, Takeda, and AbbVie; and is a member of trial steering committees for Loxo Oncology and BMS. C.U.N. has received research funding and/or consultancy fees from Abbvie, Janssen, AstraZeneca, Genmab, Beigene, Octapharma, MSD, Lilly, Takeda, CSL Behring. N.N.S. reports participation on advisory boards and/or consultancy for Gilead‐Kite, BMS‐Juno, Miltenyi Biomedicine, Lilly Oncology, Incyte, Novartis, Seattle Genetics, Janssen, AbbVie, Cargo, Beigene, and Galapagos. He has research funding, travel support, and honoraria from Lilly Oncology, Genentech, and Miltenyi Biomedicine. In addition, N.N.S. is on a scientific advisory board for Tundra Therapeutics. M.D. reports participation on advisory boards for AbbVie, Astra Zeneca, Beigene, BMS/Celgene, Gilead/Kite, Janssen, Lilly/Loxo, Novartis, Roche. He has research funding from AbbVie, Bayer, BMS/Celgene, Gilead/Kite, Janssen, Roche, and honoraria from Astra Zeneca, Beigene, Gilead/Kite, Janssen, Lilly, Novartis, Roche. F.C. reports participation on advisory boards and/or consultancy for Lilly and Astra Zeneca. T.A.E., C.U.N., N.N.S., M.D., and F.C. received research honoraria from AXDEV Global for their contribution to the study design, review of research tools, and interpretation of the findings from this study. S.Z. and B.G. are employees of Eli Lilly and Company.

## Supporting information


**Supporting Information: A.** Checklist for Reporting Of Survey Studies (CROSS).

**Supporting Information: B.** Survey structure and questions.

## Data Availability

Data used in this study is available in aggregated form upon reasonable request by contacting the corresponding author.
