# Supplementary material for: Treatment, Monitoring, and Management of Patients With Relapsed/Refractory Chronic Lymphocytic Leukemia and Mantle Cell Lymphoma: Evidence From a Multi‐Country Needs Assessment
Source: Cancer Rep (Hoboken). 2026 Aug 13;9(8):e70655. doi: 10.1002/cnr2.70655 (PMC13473176; doi:10.1002/cnr2.70655)
Supplement: Supplementary file 1 [file CNR2-9-e70655-s001.docx]

**Supplementary Information B. Survey structure and questions**

**Note**: *The survey below shows the general structure of the survey that was used. It includes all questions which after analysis, were considered aligned with the main focus of the manuscript (managing and monitoring treatment, engaging patients in shared decision-making) and relevant to include by the co-authors.*

Hello and Welcome! Thank for your interest in this study. To confirm your eligibility, AXDEV must ask you some questions about yourself and your practice. You may stop participating in this screening process at any time. Please note that participation is limited based on categories and the study will be closed once we have reached the required number of participants. You may only participate once.

1. **Where is your practice located?**
a. Brazil
b. France
c. Germany
d. United Kingdom
e. United States
f. None of the above 🡪 Screen out
2. **Are you an actively practicing physician (i.e., 50% or more of your professional time is spent caring for patients, and you are not retired)?**
   a. Yes
   b. No 🡪 Screen out
3. **Which of the following best describes your specialty?**
   a. Hematologist
   b. Hemato-Oncologist
   c. Oncologist
   d. Other 🡪 Screen out
4. **How many unique patients with chronic lymphocytic leukemia (CLL) have you personally treated in the past two years?**

Enter number (up to 9999) 🡪 Screen out if less than 5

5. **How many unique patients with mantle cell lymphoma (MCL) have you treated in the past two years?**

Enter number (up to 9999) 🡪 Screen out if less than 2

6. **Do you have patients with CLL or MCL who are on treatment with Bruton's tyrosine kinase (BTK) inhibitors (e.g., ibrutinib,** **acalabrutinib)?**
   a. Yes
   b. No 🡪 Screen out
7. **How many years have you been working in the field of hematology or hemato-oncology (after completion of residency)?**
a. Less than 2 years 🡪 Screen out
b. 2-5 years
c. 6-10 years
d. 11-20 years
e. 21 years or more
8. (if 1=a/b/c Brazil/France/Germany ) **Which of the following best describes your main work setting?**
a. An academic-based setting🡪 screen out
b. A community-based setting
9. (if 1=d= United Kingdom) **Which of the following best describes your main work setting?**
   a. Tertiary Cancer Centre
   b. District General Hospital
   c. Private hospital/clinic
   d. Hospital (academic-based or academic-affiliated) 🡪 screen out
   e. A solo practice or single-specialty clinic or center Academic-based or academic-affiliated clinic or centre 🡪 screen out
   f. Other (please specify)
10. (if 1=e= United States) Which of the following best describes your main work setting?
a. A solo practice or single-specialty clinic or center outside a hospital
b. A solo practice or single-specialty clinic or center (hematology or hemato-oncology) outside a hospital
c. A solo practice or single-specialty clinic or center (hematology or hemato-oncology) outside a hospital
d. Multi-specialty clinic or center outside a hospital
e. Hospital (academic-based or academic-affiliated)
f. A solo practice or single-specialty clinic or center (academic-based or academic-affiliated)
g. Public hospital (community-based – non-academic-affiliated)
h. Private hospital / clinic
i. Government medicine (e.g. Veteran’s Affairs)
j. Other

Insert IRB-approved consent here

Section 1: Introduction

“This survey will take approximately 15-20 minutes to complete. Your responses will inform future education. Before starting the survey, please ensure you can dedicate sufficient time to provide honest and accurate responses. **If need be, you may start the survey and complete it later**. Thank you for your participation.”

Section 2 - CLL

Section 2.1 – Knowledge

Question 1 CLL (to all). **For each item listed below, please rate your current level of knowledge based on what is expected of your professional role in the care of patients with** **refractory/relapsed CLL.**

Note: If item is NOT relevant to your current professional role, please select Not relevant (“NR”).

1=No knowledge at all; 2=Basic knowledge; 3=Intermediate knowledge; 4=Advanced knowledge; 5=Expert knowledge; NR=Not relevant

a. Side effect profiles of CD20 monoclonal antibodies (e.g., rituximab, obinutuzumab)
b. Side effect profiles of more recently (after 2018) approved BTK inhibitors (e.g., acalabrutinib)
c. Side effect profiles of non-covalent BTK inhibitor

Section 2.2 Skills

Question 2 CLL (to all). **For each item listed below, please rate your current level of skill according to what is expected of your professional role in the care of patients with refractory/relapsed CLL.**

Note: If item is NOT relevant to your current professional role, please select Not relevant (“NR”).

1=No skills at all; 2=Basic skills; 3=Intermediate skills; 4=Advanced skills; 5=Expert skills; NR=Not relevant

a. Identifying patients at higher risk for progression and using stratification to plan and manage treatment
b. Monitor and manage side effects from BCL-2 inhibitors
c. Monitor and manage side effects from BTK inhibitors
d. Adjusting treatment approach based on patients’ tolerance
e. Incorporating recommendations from guidelines to adjust treatment
f. Weighting recommendations from guidelines and patient preferences to select new treatment in case of intolerance or toxicity

Section 2.3 Confidence

Question 3 CLL (to all). **For each item listed below, please rate your current level of confidence performing specific tasks as part of your professional role in the care of patients with CLL.**

Note: If item is NOT relevant to your current professional role, please select Not relevant (“NR”).

1=Not confident at all, 2=Slightly confident, 3=Somewhat confident, 4=Confident, 5=Very confident, NR=Not relevant

a. Selecting treatment in consideration of potential side effects and toxicities

Section 2.4 Agreement

Question 4 (to all). **Please rate your level of agreement with each of the following statements:**

Note: If you are unsure and/or not informed enough to form an opinion, please select “Unsure”

0= Unsure - not informed enough to form an opinion; 1=Strongly disagree; 2=Disagree; 3=Slightly disagree; 4=Slightly Agree; 5=Agree; 6=Strongly agree

a. BCL-2 inhibitors and BTK inhibitors have similar side effect profile, but different OS for patients with CLL

Question 6 (to all). **Which of the following reflect more closely your perspective on the risk/benefit ratio of BTK inhibitors among your patients on anticoagulation?**

a. Risks almost always outweigh benefits
b. Risks outweigh benefits most of the time
c. Risks outweigh benefits just as often as benefits outweigh risks
d. Benefits outweigh risks most of the time
e. Benefits almost always outweigh risks

Section 2.5 –System Challenges

Question 8 (to all). **How frequently do the following aspects of a patients’ situation or context beyond their disease state influence you to recommend targeted therapies?**

0= unsure 1=Never; 2=Rarely; 3=Sometimes; 4=Often; 5=Always

Note: If you are unsure and/or not informed enough to form an opinion, please select “Unsure”

a. Patients’ likelihood of adhering to the treatment
b. Concerns about managing side effects

Section 2.6 Cases

Section 3 MCL

Section 3.1 Knowledge

Section 3.2 – Skills

Question 12 (to all). **For each item listed below, please rate your current level of skill according to what is expected of your professional role in the care of patients with refractory/relapsed MCL.**

Note: If item is NOT relevant to your current professional role, please select Not relevant (“NR”).

1=No skills at all; 2=Basic skills; 3=Intermediate skills; 4=Advanced skills; 5=Expert skills; NR=Not relevant

a. Monitor and manage side effects from BTK inhibitors
b. Monitor and manage side effects from CAR-T cell therapy
c. Incorporating recommendations from guidelines to adjust treatment
d. Weighting recommendations from guidelines and patient preferences to select new treatment in case of intolerance or toxicity

Section 3.3 – Confidence

Question 13 (to all). **For each item listed below, please rate your current level of confidence performing specific tasks as part of your professional role in the care of patients with refractory/relapse MCL.**

Note: If item is NOT relevant to your current professional role, please select Not relevant (“NR”).

1=Not confident at all, 2=Slightly confident, 3=Somewhat confident, 4=Confident, 5=Very confident, NR=Not relevant

a. Selecting treatment in consideration of potential side effects and toxicities

Section 3.4 – Agreement

Section 4: Both CLL and MCL

Question 17 both (to all). **For each item listed below, please rate your current level of confidence performing specific tasks as part of your professional role in the care of patients with refractory/relapse CLL and MCL.**

Note: If item is NOT relevant to your current professional role, please select Not relevant (“NR”).

1=Not confident at all, 2=Slightly confident, 3=Somewhat confident, 4=Confident, 5=Very confident, NR=Not relevant

a. Engaging patients in discussion of risk and benefits of therapy, including side effects, overall survival (OS), and progression free survival (PFS)
b. Educating patients to help them distinguish what is a lack of response to treatment vs. a manageable side effect
c. Recognizing BTK inhibitor resistance

Question 18 (to all). **Please rate your level of agreement with each of the following statements:**

Note: If you are unsure and/or not informed enough to form an opinion, please select “Unsure”

0= Unsure - not informed enough to form an opinion; 1=Strongly disagree; 2=Disagree; 3=Slightly disagree; 4=Slightly Agree; 5=Agree; 6=Strongly agree

a. Non-covalent BTK inhibitors may have a better side effect profile, but they cannot overcome resistance

Question 19 (to all). **How often do you perform the following actions when caring for patients with CLL and MCL?**

Note: If a given item is NOT relevant to your current professional role, please select Not relevant (“NR”).

1=Never; 2=Rarely, in few cases; 3=Sometimes 4=Often; 5=Always; NR=Not relevant

a. Ask patients their preferences for therapy

Section 5 – Conclusion (to all)

Question 20 (to all). **Please share any final comments you may have regarding educational needs in the treatment and management of patients with refractory/relapse CLL/MCL or regarding this survey.**

[open field]

Section 6 – Thank you (to all)

Thank you for completing this survey. We appreciate your insight and time.
